# Supplementary material for: Emulated Clinical Trials from Longitudinal Real-World Data Efficiently Identify Candidates for Neurological Disease Modification: Examples from Parkinson’s Disease
Source: Front Pharmacol. 2021 Apr 22;12:631584. doi: 10.3389/fphar.2021.631584 (PMC8100658; doi:10.3389/fphar.2021.631584)
Supplement: Supplementary file 1 [file datasheet1.zip › supplementary material/Tables S1-S9.docx]

Supplemental Material for “Emulated Clinical Trials from Longitudinal Real-World Data Efficiently Identify Candidates for Neurological Disease Modification: Examples from Parkinson's Disease”

Table S1. ICD codes for PD cohort definition

| **Type** | **System** | **Code** | **Name** |
| --- | --- | --- | --- |
| Inclusion | icd9 | 3320 | Paralysis agitans |
|  | icd10 | G20 | Parkinson's disease |
| Exclusion | icd9 | 3316 | Corticobasal degeneration |
|  | icd9 | 3321 | Secondary parkinsonism |
|  | icd9 | 3330 | Other degenerative diseases of the basal ganglia |
|  | icd10 | G21 | Secondary parkinsonism |
|  | icd10 | G231 | Progressive supranuclear ophthalmoplegia [Steele-Richardson-Olszewski] |
|  | icd10 | G3185 | Corticobasal degeneration |

Table S2. PD-indicated drugs and their corresponding ATC class names. The list below has been compiled by a domain expert based on the following sources: National Drug File – Reference Terminology (NDF-RT), Anatomical Therapeutic Chemical Classification System (ATC), and DrugBank *(45)*.

| **Drug** | **ATC class code(s)** | **ATC class name(s)** |
| --- | --- | --- |
| Amantadine | N04BB | Adamantane derivatives |
| Apomorphine | G04BE; N04BC | Drugs used in erectile dysfunction; dopamine agonists |
| Atropine | A03BA; S01FA | Belladonna alkaloids, tertiary amines; anticholinergics |
| Benztropine | N04AC | Ethers of tropine or tropine derivatives |
| Biperiden | N04AA | Tertiary amines |
| Bornaprine | N04AA | Tertiary amines |
| Bromocriptine | G02CB; N04BC | Prolactine inhibitors; dopamine agonists |
| Budipine | N04BX | Other dopaminergic agents |
| Cabergoline | G02CB; N04BC | Prolactine inhibitors; dopamine agonists |
| Carbidopa | N/A | N/A |
| Dexetimide | N04AA | Tertiary amines |
| Dihydroergocryptine | N04BC | Dopamine agonists |
| Entacapone | N04BX | Other dopaminergic agents |
| Hyoscyamine | A03BA | Belladonna alkaloids, tertiary amines |
| Levodopa | N04BA | Dopa and dopa derivatives |
| Methixene | N04AA | Tertiary amines |
| Orphenadrine | N04AB; M03BC | Ethers chemically close to antihistamines; ethers, chemically close to antihistamines |
| Pergolide | N04BC | Dopamine agonists |
| Piribedil | N04BC | Dopamine agonists |
| Pramipexole | N04BC | Dopamine agonists |
| Procyclidine | N04AA | Tertiary amines |
| Profenamine | N04AA | Tertiary amines |
| Rasagiline | N04BD | Monoamine oxidase B inhibitors |
| Ropinirole | N04BC | Dopamine agonists |
| Rotigotine | N04BC | Dopamine agonists |
| Selegiline | N04BD | Monoamine oxidase B inhibitors |
| Tolcapone | N04BX | Other dopaminergic agents |
| Trihexyphenidyl | N04AA | Tertiary amines |
| Tropatepine | N04AA | Tertiary amines |

Table S3. PD outcome definitions.

| Outcome | System | Code | Name |
| --- | --- | --- | --- |
| Fall | ICD9 | E88 | Accidental falls |
|  | ICD10 | W0, W1 | Falls |
| Psychosis | ICD9 | 292 | Drug-induced mental disorders |
|  |  | 297 | Delusional disorders |
|  |  | 298 | Other nonorganic psychoses |
|  | ICD10 | F2 | Schizophrenia, schizotypal and delusional disorders |
| Dementia | ICD9 | 294 | Persistent mental disorders due to conditions classified elsewhere |
|  |  | 331 | Other cerebral degenerations |
|  | ICD10 | F01 | Vascular dementia |
|  |  | F02 | Dementia in other diseases classified elsewhere |
|  |  | F03 | Unspecified dementia |
|  |  | G30 | Alzheimer disease |

Table S4. Rasagiline cohort characteristics. Shown are the mean, standard deviation (in parentheses), and the first, second (median), and third quartile (in brackets).

|  | MarketScan | | Explorys |
| --- | --- | --- | --- |
| Patient count | 3,094 | | 1,988 |
| Patient timeline [years] |  | |  |
| Total | 4.1 (1.0) [3.0; 4.6; 5.0] | | 11.2 (5.1) [7.0; 10.6; 14.8] |
| Before index date | 2.5 (1.0) [1.6; 2.4; 3.2] | | 7.3 (4.8) [3.3; 6.3; 10.6] |
| After index date | 1.6 (1.0) [0.8; 1.5; 2.4] | | 3.9 (2.5) [2.0; 3.5; 5.4] |
| No. of unique prescribed drugs | 15.6 (9.3) [9.0; 14.0; 21.0] | | 20.7 (18.2) [7.0; 15.0; 29.0] |
| Insurance |  | |  |
| Medicare, Medicaid, other public | 60% | | 80% |
| Commercial, private only | 40% | | 18% |
| Other or unknown | 0% | | 2% |
| Baseline characteristics (during ≤1 year index date) | |  | |
| Age at index date | 68.5 (8.5) [61.8; 66.2; 74.7] | | 70.7 (7.7) [64.8; 70.4; 76.4] |
| Women | 38% | | 37% |
| Charlson's Comorbidity Index | 1.1 (1.5) [0.0; 0.0; 2.0] | | 0.6 (1.2) [0.0; 0.0; 1.0] |
| PD related diagnoses | |  | |
| Falls | 3% | | 4% |
| Psychosis | 1% | | 1% |
| Follow-up characteristics (during ≤2 years following index date) | | | |
| PD Progression | |  | |
| Dementia | 15%^†^ (11%) | | 12%^†^ (11%) |
| Charlson's Comorbidity Index | 1.4 (1.9) [0.0; 1.0; 2.0] | | 1.1 (1.8) [0.0; 0.0; 2.0] |

† 15% and 12% are Kaplan–Meier estimates in MarketScan and Explorys resp., which adjust for censoring.

Table S5. Zolpidem cohort characteristics. Shown are the mean, standard deviation (in parentheses), and the first, second (median), and third quartile (in brackets).

|  | MarketScan | Explorys |
| --- | --- | --- |
| Patient count | 847 | 1,828 |
| Patient timeline [years] |  |  |
| Total | 4.0 (1.1) [3.0; 4.1; 5.0] | - 1. (5.1) [7.2; 11.1; 15.2] |
| Before index date | 2.2 (1.0) [1.4; 2.0; 2.8] | - 1. (4.7) [3.6; 6.7; 10.7] |
| After index date | 1.8 (1.1) [0.9; 1.6; 2.6] | 3.9 (2.6) [1.8; 3.4; 5.4] |
| No. of unique prescribed drugs | 25.1 (12.7) [16.0; 23.0; 32.0] | 40.3 (25.8) [20.0; 35.0; 55.0] |
| Insurance |  |  |
| Medicare, Medicaid, other public | 77% | 83% |
| Commercial, private only | 23% | 9% |
| Other or unknown | 0% | 8% |
| Baseline characteristics (during ≤1 year before index date) | |  |
| Age at index date | 71.4 (10.1) [62.6; 70.0; 79.5] | - 1. (7.8) [67.8; 74.8; 80.2] |
| Women | 43% | 45% |
| Charlson's Comorbidity Index | 2.1 (2.3) [0.0; 1.0; 3.0] | 1.7 (2.1) [0.0; 1.0; 3.0] |
| PD related diagnoses | |  |
| Falls | 8% | 11% |
| Psychosis | 5% | 4% |
| Follow-up characteristics (during ≤2 years following index date) | | |
| PD Progression | |  |
| Dementia | 26%^†^ (20%) | 23%^†^ (21%) |
| Charlson's Comorbidity Index | 2.8 (2.9) [0.0; 2.0; 4.0] | 3.0 (3.0) [1.0; 2.0; 5.0] |

† 26% and 23% are Kaplan–Meier estimates in MarketScan and Explorys resp., which adjust for censoring.

Table S6. N04 cohort characteristics. Shown are the mean, standard deviation (in parentheses), and the first, second (median), and third quartile (in brackets).

|  | MarketScan | Explorys |
| --- | --- | --- |
| Patient count | 10,289 | 12,408 |
| Patient timeline [years] |  |  |
| Total | 4.1 (1.0) [3.1; 4.8; 5.0] | 10.9 (5.1) [6.8; 10.5; 14.6] |
| Before index date | 2.5 (1.1) [1.6; 2.3; 3.3] | 7.5 (4.7) [3.6; 6.5; 10.6] |
| After index date | 1.6 (1.0) [0.8; 1.4; 2.4] | 3.5 (2.6) [1.5; 2.8; 4.8] |
| No. of unique prescribed drugs | 17.4 (10.4) [10.0; 16.0; 23.0] | 18.3 (17.8) [5.0; 13.0; 25.0] |
| Insurance |  |  |
| Medicare, Medicaid, other public | 79% | 82% |
| Commercial, private only | 21% | 12% |
| Other or unknown | 0% | 6% |
| Baseline characteristics (during ≤1 year before index date) | |  |
| Age at first diagnosis | 73.3 (9.8) [64.6; 73.6; 81.1] | 73.7 (7.9) [67.9; 74.5; 79.9] |
| Women | 42% | 44% |
| Charlson's Comorbidity Index | 1.7 (2.0) [0.0; 1.0; 3.0] | 0.8 (1.3) [0.0; 0.0; 1.0] |
| PD related diagnoses | |  |
| Falls | 5% | 4% |
| Psychosis | 3% | 1% |
| Follow-up characteristics (during ≤2 years following index date) | | |
| PD progression | |  |
| Dementia | 28%^†^ (21%) | 19%^†^ (17%) |
| Charlson's Comorbidity Index | 2.2 (2.5) [0.0; 1.0; 3.0] | 1.6 (2.1) [0.0; 1.0; 2.0] |

† 28% and 19% are Kaplan–Meier estimates in MarketScan and Explorys resp., which adjust for censoring.

Table S7. N05 cohort characteristics. Shown are the mean, standard deviation (in parentheses), and the first, second (median), and third quartile (in brackets).

|  | MarketScan | Explorys |
| --- | --- | --- |
| Patient count | 3,116 | 9,067 |
| Patient timeline [years] |  |  |
| Total | 4.0 (1.1) [3.0; 4.2; 5.0] | 10.4 (5.0) [6.5; 9.6; 14.1] |
| Before index date | 2.3 (1.0) [1.5; 2.1; 3.0] | 7.1 (4.6) [3.4; 6.1; 10.1] |
| After index date | 1.7 (1.1) [0.8; 1.5; 2.6] | 3.3 (2.4) [1.5; 2.8; 4.5] |
| No. of unique prescribed drugs | 19.4 (10.0) [12.0; 18.0; 25.0] | 24.4 (17.8) [11.0; 20.0; 33.0] |
| Insurance |  |  |
| Medicare, Medicaid, other public | 83% | 84% |
| Commercial, private only | 17% | 9% |
| Other or unknown | 0% | 7% |
| Baseline characteristics (during ≤1 year before index date) | |  |
| Age at first diagnosis | 74.0 (9.9) [65.1; 74.4; 82.1] | (7.9) [68.9; 75.4; 80.8] |
| Women | 48% | 45% |
| Charlson's Comorbidity Index | 1.8 (2.1) [0.0; 1.0; 3.0] | 1.1 (1.7) [0.0; 0.0; 2.0] |
| PD related diagnoses | |  |
| Falls | 6% | 10% |
| Psychosis | 7% | 4% |
| Follow-up characteristics (during ≤2 years following index date) | |  |
| PD progression | |  |
| Dementia | 38%^†^ (30%) | 29%^†^ (27%) |
| Charlson's Comorbidity Index | 2.4 (2.7) [0.0; 2.0; 4.0] | 2.3 (2.6) [0.0; 2.0; 4.0] |

† 38% and 29% are Kaplan–Meier estimates in MarketScan and Explorys resp., which adjust for censoring.

Table S8. Estimated effects on dementia onset for emulated RCTs involving rasagiline and its encompassing ATC classes. For composition of treatment and control cohorts, see Table S10. Beneficial effect is highlighted in green and non-beneficial effect is highlighted in red.

|  |  | **Control cohort** | | | | | | | |
| --- | --- | --- | --- | --- | --- | --- | --- | --- | --- |
|  |  | N04BD | | N04B | | N04 | | N | |
|  |  | **Estimated effects** | | | | | | | |
|  |  | Weight balancing | Outcome model | Weight balancing | Outcome model | Weight balancing | Outcome model | Weight balancing | Outcome model |
|  |  | **MarketScan** | | | | | | | |
| Treatment cohort | Rasagiline | -0.009 | -0.06 | -0.09 | -0.07 | -0.09 | -0.07 | -0.09 | -0.07 |
|  | N04BD |  |  | -0.08 | -0.06 | -0.08 | -0.06 | -0.08 | -0.06 |
|  | N04B |  |  |  |  | 0.13 | -0.08 | Failed to balance cohorts | |
|  | N04 |  |  |  |  |  |  | Failed to balance cohorts | |
|  |  | **Explorys** | | | | | | | |
| Treatment cohort | Rasagiline | -0.06 | -0.08 | -0.09 | -0.07 | -0.08 | -0.07 | -0.09 | -0.09 |
|  | N04BD |  |  | -0.11 | -0.11 | -0.11 | -0.1 | -0.08 | -0.08 |
|  | N04B |  |  |  |  | Failed to balance cohorts | | -0.00013 | -0.11 |
|  | N04 |  |  |  |  |  |  | -0.01 | -0.1 |

Table S9. Estimated effects on dementia onset for emulated RCTs involving zolpidem and its encompassing ATC classes. For composition of treatment and control cohorts, see Table S10. Beneficial effect is highlighted in green and non-beneficial effect is highlighted in red.

|  |  | **Control cohort** | | | | | | | |
| --- | --- | --- | --- | --- | --- | --- | --- | --- | --- |
|  |  | N05CF | | N05C | | N05 | | N | |
|  |  | **Estimated effects** | | | | | | | |
|  |  | Weight balancing | Outcome model | Weight balancing | Outcome model | Weight balancing | Outcome model | Weight balancing | Outcome model |
|  |  | MarketScan | | | | | | | |
| Treatment cohort | Zolpidem | -0.03 | -0.1 | -0.13 | -0.13 | -0.12 | -0.1 | -0.09 | -0.04 |
|  | N05CF |  |  | -0.13 | -0.12 | -0.13 | -0.12 | -0.08 | -0.05 |
|  | N05C |  |  |  |  | -0.06 | -0.07 | -0.05 | -0.006 |
|  | N05 |  |  |  |  |  |  | 0.03 | 0.02 |
|  |  | Explorys | | | | | | | |
| Treatment cohort | Zolpidem | Control cohort too small | | 0.01 | 0.006 | -0.08 | -0.08 | -0.02 | -0.01 |
|  | N05CF |  |  | 0.03 | 0.01 | -0.06 | -0.07 | -0.02 | -0.01 |
|  | N05C |  |  |  |  | -0.12 | -0.11 | -0.06 | -0.03 |
|  | N05 |  |  |  |  |  |  | 0.07 | 0.02 |
